# The relation between cerebral small vessel function and white matter microstructure in monogenic and sporadic small vessel disease - the ZOOM@SVDs study

**DOI:** 10.1016/j.cccb.2025.100383

**Published:** 2025-03-24

**Authors:** Naomi Vlegels, Hilde van den Brink, Anna Kopczak, Tine Arts, Stanley D.T. Pham, Jeroen C.W. Siero, Benno Gesierich, Alberto De Luca, Marco Duering, Jaco J.M. Zwanenburg, Martin Dichgans, Geert Jan Biessels

**Affiliations:** a Department of Neurology and Neurosurgery, UMC Utrecht Brain Center, University Medical Center Utrecht, Utrecht University, Utrecht, The Netherlands; b Institute for Stroke and Dementia Research, University Hospital, LMU Munich, Munich, Germany; c Translational Neuroimaging Group, Center for Image Sciences, University Medical Center Utrecht, Utrecht, The Netherlands; d Spinoza Centre for Neuroimaging Amsterdam, Amsterdam, The Netherlands; e Medical Image Analysis Center (MIAC) and Department of Biomedical Engineering, University of Basel, Basel, Switzerland; f Image Sciences Institute, Division Imaging and Oncology, University Medical Center Utrecht, Utrecht, The Netherlands; g Munich Cluster for Systems Neurology (SyNergy), Munich, Germany; h German Center for Neurodegenerative Disease (DZNE), Munich, Germany

**Keywords:** CADASIL, Cerebral small vessel disease, Small vessel function, Ultra-high field strength MRI, White matter microstructure

## Abstract

In cerebral small vessel disease (cSVD), vascular dysfunction has been associated with cSVD-lesions across the brain. Here we further explore the relation between vascular dysfunction and cSVD-related brain injury. We tested two hypotheses: (1) that complementary measures of abnormal small vessel function relate to decreased white matter integrity, and (2) that local variance in vascular dysfunction relates to local variance in white matter integrity within individual patients.

We included 23 patients with monogenic cSVD (i.e. CADASIL) and 46 patients with sporadic cSVD. With whole-brain analyses, we tested if small vessel flow velocity and reactivity measures from 7T-MRI were associated with global peak-width-of-skeletonized-mean-diffusivity (PSMD). We also tested voxel-wise correlations between reactivity to hypercapnia and mean diffusivity (MD) in white matter.

Whole-brain analyses showed a negative association between blood flow velocity and PSMD for the perforating arteries in the centrum semiovale in CADASIL (*p* = 0.04) and in the basal ganglia in sporadic cSVD (*p* = 0.002). Global white matter reactivity to hypercapnia was not associated with PSMD. Within patients, both in CADASIL and sporadic cSVD, we observed significant voxel-wise negative correlations for endothelial-independent vascular reactivity and MD in the white matter.

These findings confirm our hypothesis that small vessel dysfunction in patients with cSVD is associated with microstructural white matter alterations, also at voxel level. The latter may reflect a direct relationship between local small vessel dysfunction and tissue injury.

## Highlights

• Small vessel dysfunction is associated with lower white matter integrity in cSVD.
• Voxel-wise observations provide a lead towards possible causality.
• Observations were similar in patients with monogenic and sporadic cSVD.

## 1 Introduction

Cerebral small vessel disease (cSVD) is a major cause of stroke and dementia [1,2]. With MRI, cSVD is mostly studied through markers of parenchymal injury (e.g. white matter hyperintensities (WMH), lacunes, cerebral microbleeds) [3]. The processes that underlie the formation of these lesions likely involve disturbances at the level of the cerebral small vessels. Due to their small size, these vessels are difficult to probe *in vivo*. Previous studies on the small vessels in cSVD therefore mostly involved neuropathological assessment of autopsy material, showing loss of smooth muscle cells, thickening of the vessel walls and luminal narrowing in cerebral arterioles, but also abnormalities in capillaries and venules [2]. Over the past years, these neuropathological observations have been complemented by functional vascular measures using MRI [4]. This included studies at common field strengths (i.e., up to 3T-MRI), showing that decreased vascular reactivity related to increased WMH burden [[5], [6], [7], [8], [9], [10], [11], [12]]. With technological advancements in 7T-MRI, we can now assess small vessel flow velocity and pulsatility index in cerebral perforating arteries as well as (small) vessel reactivity with a sensitivity and temporal and spatial resolution that was not possible before *in vivo* in humans [13]. These complementary aspects of small vessel function can be assessed in different vessel populations throughout the brain, providing insights into underlying disease mechanisms of cSVD in different parts of the vascular tree. We recently showed abnormalities of small vessel function on 7T-MRI in patients with CADASIL and sporadic cSVD, indicative of regional abnormalities in arteriolar stiffness and reactivity [14,15]. Specifically, we observed lower blood flow velocity and higher pulsatility index in perforating arteries in the centrum semiovale in CADASIL and in perforating arteries in the basal ganglia in sporadic cSVD. In addition, small vessel reactivity to a short visual stimulus was lower in patients with CADASIL and shortened in sporadic cSVD and reactivity to a hypercapnic stimulus was decreased in WMH versus NAWM in both patient groups (Summary published results in Supplementary Table 1 for reference). These abnormalities of small vessel function were also related to higher WMH volume and higher lacune count [14,15].

In the current study, we further explore the relation between small vessel dysfunction and cSVD-related brain injury using diffusion MRI. Diffusion MRI-based measures of the white matter microstructure are currently the most sensitive method for studying tissue injury in cSVD [16,17]. Diffusion MRI quantifies the diffusion properties of water molecules in brain tissue and is thereby highly sensitive in detecting subtle tissue alterations, also regionally with high spatial resolution. Moreover, cSVD-related diffusion alterations are associated with clinical deficits and typically outperform conventional MRI markers in terms of strength of this association [17,18]. Diffusion alterations in cSVD can be assessed with robust global measures such as *“peak width of skeletonized mean diffusivity”* (PSMD), a sensitive measure of white matter microstructure [17], but also locally in the white matter at a voxel-level. With voxel-level measurements, a relation between local variance in vascular dysfunction and local variance in white matter integrity can be assessed.

This study aims to test the hypotheses that (1) complementary measures of abnormal small vessel function relate to decreased white matter integrity, and that (2) local variance in vascular dysfunction relates to local variance in white matter integrity within individual patients. To address the first hypothesis, we performed whole-brain analyses where we related the aforementioned measures of small vessel dysfunction on 7T-MRI to PSMD as a sensitive and robust measure of whole-brain white matter microstructure. To address the second hypothesis, we performed voxel-wise analyses in which we related local small vessel reactivity to local mean diffusivity throughout the brain. We tested our hypotheses both in patients with CADASIL and in patients with sporadic cSVD, to assess if observations are consistent across multiple forms of cSVD, and included controls as a frame of reference.

## 2 Materials and methods

### 2.1 Participants and study procedure

Patients with monogenic and sporadic cSVD were recruited through the ZOOM@SVDs study, a prospective observational cohort study [13], at the Institute of Stroke and Dementia Research at Ludwig-Maximilians-Universität (LMU) Munich, Germany, and the University Medical Center Utrecht (UMCU) in The Netherlands. Detailed inclusion and exclusion criteria and study procedures are published in the design paper of ZOOM@SVDs [13], but a short description can be found below:

• Monogenic cSVD: At LMU, a tertiary national referral centre for patients with CADASIL in Germany, 23 patients with CADASIL and 13 age- and sex-matched reference participants were recruited. CADASIL was either confirmed by molecular genetic testing (*n* = 20) or by skin biopsy (*n* = 3). Reference participants without cSVD (defined as no history of stroke or of cognitive complaints for which the person has previously sought medical advice, and no so-called “silent” cSVD, i.e. Fazekas <2 and no lacunes, on the study MRI) were recruited among partners or relatives of the patients and through advertisement. There were no screen failures. All participants underwent clinical assessment and 3T brain MRI at LMU and travelled to the UMCU to undergo 7T brain MRI.
• Sporadic cSVD: At the stroke and memory clinics of the UMCU and referring centres, 54 patients with symptomatic sporadic cSVD and 28 age- and sex-matched reference participants were recruited. Symptomatic sporadic cSVD was defined as having a history of clinical lacunar stroke with a corresponding acute lesion on MRI or CT in the last 5 years, or having cognitive impairment with confluent WMH on MRI (Fazekas ≥ 2). Reference participants without cSVD were recruited among partners or relatives of the patients and through advertisement. There were 3 patients for whom we could not confirm a small subcortical infarct on the study MRI, 3 reference participants were excluded because of signs of silent cSVD on the study MRI and 1 reference participant was excluded because of objective cognitive impairment. This left 51 included patients and 24 included reference participants. These participants underwent clinical assessment, 3T and 7T brain MRI at the UMCU.

For the current study, we only included patients and reference participants with at least one available small vessel function measure on 7T-MRI and available diffusion MRI. We could include all patients with CADASIL (*n* = 23) and their reference participants (*n* = 13). We had to exclude 5 patients with sporadic cSVD and 2 of their reference participants due to missing small vessel function measures and 1 of the reference participants due to a failed diffusion scan, leaving 46 patients with sporadic cSVD and 21 reference participants for this study.

The Medical Ethics Review Committees of LMU and UMCU both approved the study, which was conducted in accordance with the declaration of Helsinki and the European law of General Data Protection Regulation. Written informed consent was obtained from all participants prior to enrolment in the study. The raw research data includes confidential information and can therefore not be shared in a data deposit. Derived data can be obtained from the corresponding author upon reasonable request.

### 2.2 Brain MRI acquisition

At LMU, 3T brain MRI was acquired in patients with CADASIL and reference participants on a Siemens Magnetom Skyra 3T scanner with a 64-channel head/neck coil. At UMCU, a Philips Achieva 3T scanner with an 8-channel SENSE head coil was used for the 3T brain MRI in patients with sporadic cSVD and reference participants. The scan protocol and acquisition parameters have been previously published [13] and included a 3D T1-weighted gradient echo, a 3D T2*-weighted gradient echo, a 3D fluid-attenuated inversion recovery (FLAIR), and a diffusion-weighted MRI scan (LMU: voxel size 2 × 2 × 2m^3^, TR/TE: 3800/104.8 ms, b-values: 0, 1000 and 2000 s/mm^2^, 90 diffusion directions (30 for *b* = 1000 s/mm^2^, 60 for *b* = 2000 s/mm^2^); UMCU: voxel size 2.5 × 2.5 × 2.5mm^3^, TR/TE: 8185/73 ms, b-values 0 and 1200 s/mm^2^, 45 diffusion directions).

All participants underwent a 7T brain MRI on the same Philips 7T scanner (Philips Healthcare, Best, The Netherlands) using a 32-channel receive head coil in combination with a quadrature transmit coil (Nova Medical, MA, USA). The scan protocol and acquisition parameters are published elsewhere [13] and included 2D-Qflow sequences to assess blood flow velocity and pulsatility index in perforating arteries in the basal ganglia and centrum semiovale as well as blood oxygenation-level dependent (BOLD) sequences to assess endothelial-dependent and -independent vascular reactivity in response to a visual stimulus and hypercapnic challenge, respectively.

### 2.3 Conventional cSVD markers and brain volumetrics

Lacunes (on T1-weighted and FLAIR) and microbleeds (on T2*-weighted) were manually rated according to the STRIVE-criteria [3]. Volumetric measures and masks of WMH, intracranial volume, total brain volume, white matter and grey matter were acquired as previously published [13].

### 2.4 Small vessel function measures

Three complementary measures of small vessel function in different small vessel populations were acquired on 7T brain MRI. A detailed description of these measures [13], as well as the processing pipelines [14], is provided elsewhere. In short:

1. Given the high spatial resolution on 7T-MRI, 2D-Qflow velocity mapping acquisitions could be acquired at the level of the basal ganglia and centrum semiovale to assess blood flow velocity in perforating arteries. Blood flow pulsatility was than calculated as (Vmax-Vmin)/Vmean, where Vmax, Vmin, and Vmean are the maximum, minimum, and mean of normalized and averaged blood flow velocity. In these cohorts with known small vessel alterations, we regard pulsatility index in perforating arteries as an indicator of perforating artery stiffness.
2. BOLD data were acquired in the visual cortex to assess endothelial-dependent (through neurovascular coupling) vascular reactivity in response to looking at a short visual stimulus. The high temporal resolution on 7T-MRI provided a very precise estimate of the average BOLD hemodynamic response function as generated by the short visual stimulus. Large draining veins were excluded from the analysis [13]. The BOLD % signal change and full-width-at-half-maximum (FWHM) were derived from the hemodynamic response function as measures of endothelial-dependent small vessel reactivity.
3. Whole-brain BOLD data were acquired to assess endothelial-independent vascular reactivity in response to a hypercapnic stimulus (i.e. breathing 6% CO_2_ in room air for 2 × 2 min). The high spatial resolution of BOLD on 7T-MRI permits regional analyses. Large draining veins were excluded from the analysis [13]. The BOLD % signal change in the cortical grey matter, total white matter and normal-appearing white matter (NAWM) were derived as measures of endothelial-independent small vessel reactivity.

### 2.5 Diffusion measures

The diffusion images for both patients with CADASIL and patients with sporadic cSVD were processed using similar pipelines. After visual inspection to exclude major artefacts, raw diffusion images were pre-processed using the MRtrix3 packages (www.mrtrix3.com) [19] and the Functional Magnetic Resonance Imaging of the Brain (FMRIB) software library (FSL), v6.0.3 [20]. First, noise and Gibbs ringing artefacts were removed (‘dwidenoise’ [[21], [22], [23]], ‘mrdegibbs’ [24], MRtrix3), followed by correction of subject motion and distortion correction (‘topup’ only for CADASIL datasets given the availability of a reverse phase encoding scan, ‘eddy’ [[25], [26], [27], [28]] for both datasets, FSL). Lastly, we corrected for bias field in the CADASIL group (ANTS [29]). Patients with CADASIL who underwent their 3T brain MRI in Munich, had a multishell diffusion MRI. After preprocessing, we only selected volumes with *b* = 0 and *b* = 1000 s/mm^2^ (‘dwiextract’, MRtrix3 [19]) and used this single shell for further processing. Using the preprocessed diffusion images, we calculated the diffusion tensors to obtain mean diffusivity (MD) maps (which were used for the voxel-wise analyses as described below) (‘dtifit’, FSL). As a marker of whole-brain microstructure of the white matter, we calculated PSMD [17]. PSMD is a sensitive and robust measure of white matter microstructure [14] and was calculated using the publicly available script (http://www.psmd-marker.com). PSMD is an index of the dispersion of mean diffusivity (MD) values across the white matter skeleton and has been described in detail before [14]. In short: to calculate PSMD, the white matter tracts are first skeletonized using tract-based spatial statistics (TBSS [30], FSL), then to avoid CSF contamination by partial volume effects, the skeleton is masked with a custom-made mask designed to exclude regions close to CSF. Lastly, with histogram analysis of MD values within the masked skeleton the peak width is calculated as the difference between the 95th and 5th percentile. PSMD was calculated both for the total white matter and for the NAWM. The NAWM mask was obtained by subtracting lesions (i.e. WMH and lacunes) from the total white matter mask and subsequent erosion of the mask.

### 2.6 Analyses

Our primary analyses, to address our hypothesis on the relation between small vessel function and white matter injury, were performed within the CADASIL and sporadic cSVD group. As a frame of reference, we performed between group analyses between each patient group and their respective control group. Differences in baseline characteristics between the patient groups and their respective reference groups were tested with independent sample *t*-tests for continuous normally distributed data, Mann Whitney U tests for non-parametric continuous data, chi-square tests for categorical data and ANOVA with age and sex correction for 7T small vessel function measures. Statistical analyses on group differences in pulsatility index were additionally corrected for mean blood flow velocity. BOLD reactivity to hypercapnia analyses were additionally corrected for change in end-tidal CO_2_ in response to hypercapnia. An overview of all measures per brain region and specification of the investigated associations can be found in Supplementary Table 2.

#### 2.6.1 Whole-brain analyses on small vessel function and diffusion alterations

To address the first research aim, i.e., to test the hypothesis that complementary measures of abnormal small vessel function relate to decreased white matter integrity, we tested the association between multiple small vessel function measures and PSMD in total white matter with univariate linear regressions within the CADASIL patient group and within the sporadic cSVD patient group. In sensitivity analyses, all analyses were repeated with NAWM PSMD, to see if the relation extends beyond visible lesions. Regression analyses were not adjusted for age, because age is strongly colinear with disease burden, particularly in CADASIL. In an additional sensitivity analysis in patients with sporadic cSVD we added age as a covariate. We refrained from multiple comparison corrections, because the different 7T small vessel function measures assess complementary aspects of vessel function in different vessel populations throughout the brain, also in light of the limited sample size and consequently statistical power.

#### 2.6.2 Voxel-wise analyses on small vessel function and diffusion alterations

To address the second research aim, i.e. to test the hypothesis that local variance in vascular dysfunction relates to local variance in white matter integrity within individual patients, we performed voxel-wise analyses both in the total WM and NAWM. For these analyses, we first registered the 3T 3D-T1 weighted images to the 7T BOLD images for each participant, using FLIRT (FSL [[31], [32], [33]]). The resulting transformation matrix was then applied to register the 3T MD map to the 7T BOLD image. All registrations were visually checked. Per participant, we then calculated the correlation coefficient between BOLD % signal change and MD across all voxels within the white matter mask. For each single participant one overall correlation coefficient was calculated in the total WM and NAWM. These per-participant voxel-wise correlations essentially eliminate the influence of possible confounders (i.e. shared risk factors for abnormal small vessel function and cerebral tissue injury). Analyses were performed in total WM and repeated in the NAWM, to test if vessel dysfunction is also already related with decreased tissue integrity in WM that appears normal on FLAIR imaging.

We performed two different analyses with these correlation coefficients. First, using a Wilcoxon signed rank test, we tested for each group if the pooled individual correlation coefficients of the BOL D% signal change with MD significantly deviated from zero. In other words, this shows if individual correlation coefficients of individual patients within each group significantly pointed to a specific direction of the correlation; i.e. worse reactivity with worse diffusion metrics. Second, we tested whether the group-level mean of individual correlation coefficients differed for patients and reference participants in both CADASIL and sporadic cSVD with Wilcoxon rank sum tests. This was done to see if the relations found were disease specific or part of normal physiology. Moreover, this analysis was repeated in the NAWM to test if disease specific relations were already apparent in tissue that otherwise still appears normal on conventional MRI. All statistical analyses were performed in R (version 4.1.3) and a significance level of *p* < 0.05 was considered significant.

## 3 Results

Characteristics of the patients with CADASIL, patients with sporadic cSVD and both matched reference groups are shown in Table 1. As reported previously [14,15] small vessel function measures, including blood flow velocity, pulsatility, and reactivity, were affected both in patients with CADASIL and sporadic cSVD, but with differences in the patterns of small vessel dysfunction (Supplementary Table 1). As expected, both patients with CADASIL and sporadic cSVD had a higher lesion load and higher PSMD (i.e. loss of white matter microstructure) than their reference groups.

**Table 1:** Characteristics of patients with CADASIL and sporadic cSVD and their respective reference groups.

	CADASIL	Reference		Sporadic cSVD	Reference	
	*n* = 23	*n* = 13	*p*	*n* = 46	*n* = 21	*p*
**Demographics**						
Age [years]	51.1 ± 10.1	46.1 ± 12.6	0.20	65.3 ± 9.4	63.3 ± 6.7	0.42
Female sex	12 (52)	6 (46)	1.00	15 (33)	8 (38)	0.87
**3T MRI cSVD markers**						
PSMD [mm^2^/s x 10^–4^]	4.1 [1.87]	2.1 [0.26]	**<0.001**	4.1 [1.97]	2.9 [0.8]	**<0.001**
WMH volume [% of ICV]	4.5 [4.4]	0.01 [0.04]	**<0.001**	1.15 [1]	0.09 [0.08]	**<0.001**
Lacune presence	13 (57)	0 (0)	**0.001**	30 (65)	0 (0)	**<0.001**
Microbleed presence	13 (57)	0 (0)	**0.001**	23 (50)	4 (9)	**0.03**
Brain volume [% of ICV]	78.3 ± 5.2	77.6 ± 3.2	0.76	69.9 ± 6.4	73.3 ± 4.3	**0.04**

Differences were tested with an independent sample *t*-test for continuous normally distributed data, Mann-Whitney U test for non-parametric continuous data (i.e. PSMD and WMH volume) and chi-square for categorical data. Data presented as M±SD, n( %) or median[IQR].

BP = blood pressure, ICV = intracranial volume, PSMD = peak width of skeletonized mean diffusivity, WMH = white matter hyperintensity.

### 3.1 Whole-brain associations between small vessel function measures and PSMD

Whole-brain analyses, aimed at addressing the first research aim, showed that within both patient groups, perforating artery flow velocity was associated with PSMD, albeit with differences in the arterioles involved. In CADASIL, lower blood flow velocity in the centrum semiovale was associated with higher PSMD (Table 2). In sporadic cSVD, this negative association was observed for blood flow velocity in the perforating arteries in the basal ganglia instead. Additionally, in this group, higher pulsatility index in the perforating arteries in the basal ganglia was associated with increased PSMD (Table 2).

**Table 2:** Linear regressions between 7T small vessel function measures and PSMD in total white matter.

	CADASIL			Sporadic cSVD		
	B	CI95	*p*	B	CI95	*p*
**2D-Qflow centrum semiovale**	*N* = 22			*N* = 46		
Blood flow velocity [cm/s]	−0.42	−0.83 – −0.03	**0.04**	0.02	−0.28 – 0.33	0.87
Pulsatility Index	−0.18	−0.63 – 0.25	0.38	0.12	−0.19 – 0.42	0.44
**2D-Qflow basal ganglia**	*N* = 21			*N* = 44		
Blood flow velocity [cm/s]	−0.23	−0.72 – 0.26	0.33	−0.45	−0.73 – −0.17	**0.002**
Pulsatility index	0.21	−0.28 – 0.70	0.38	0.31	0.01 – 0.60	**0.04**
**BOLD visual stimulus**	*N* = 19			*N* = 35		
BOLD % signal change	−0.25	−0.67 – 0.18	0.24	−0.04	−0.37 – 0.28	0.78
Full-width-at-half-maximum [s]	0.36	−0.05 – 0.76	0.08	−0.23	−0.54 – 0.08	0.14
**BOLD hypercapnic stimulus**	*N* = 17			*N* = 36		
CGM BOLD % signal change	−0.27	−0.84 – 0.31	0.35	−0.35	−0.68 – −0.03	**0.03**
NAWM BOLD % signal change	0.26	−0.33 – 0.86	0.36	−0.01	−0.36 – 0.34	0.96

B = standardized beta, BOLD = Blood oxygenation level-dependent, CGM = cortical grey matter, CI95 = 95 % confidence interval, NAWM = normal-appearing white matter, PSMD = peak width of skeletonized mean diffusivity, Qflow = quantitative flow (velocity phase contrast MRI).

Vascular reactivity to hypercapnia in the cortical grey matter was negatively associated with PSMD in patients with sporadic cSVD, but not in CADASIL, although the direction of the effect was the same (Table 2). NAWM reactivity to hypercapnia did not relate to PSMD (Table 2).

In sensitivity analyses using PSMD in the NAWM only, the above significant associations persisted (Table 3). We performed additional sensitivity analyses in patients with sporadic cSVD in which we corrected for age. In these age-corrected analyses, only the association between blood flow velocity in the perforating arteries of the basal ganglia and white matter PSMD remained significant (Standardized Beta (95% Confidence Interval) = −0.32 (−0.60 – −0.04), *p* = 0.03; Supplementary Table 3).

**Table 3:** Sensitivity analyses: linear regressions between 7T small vessel function measures and PSMD in normal appearing white matter.

	CADASIL			Sporadic cSVD		
	B	CI95	*p*	B	CI95	*p*
**2D-Qflow centrum semiovale**	*N* = 22			*N* = 46		
Blood flow velocity [cm/s]	−0.43	−0.82 – −0.05	**0.03**	−0.018	−0.34 – 0.30	0.91
WM Pulsatility Index	−0.27	−0.69 – 0.14	0.19	0.047	−0.29 – 0.38	0.78
**2D-Qflow basal ganglia**	*N* = 21			*N* = 44		
Blood flow velocity [cm/s]	−0.04	−0.54 – 0.46	0.86	−0.383	−0.68 – −0.1	**0.01**
Pulsatility index	0.09	−0.40 – 0.59	0.69	0.305	0.01 – 0.61	**0.05**
**BOLD visual stimulus**	*N* = 19			*N* = 35		
BOLD % signal change	−0.37	−0.79 – 0.05	0.08	0.017	−0.29 – 0.32	0.91
Full-width-at-half-maximum [s]	−0.15	−0.41 – 0.11	0.25	−0.154	−0.45 – 0.14	0.30
**BOLD hypercapnic stimulus**	*N* = 17			*N* = 36		
CGM BOLD % signal change	−0.37	−0.90 – 0.17	0.16	−0.36	−0.69 – −0.03	**0.03**
NAWM BOLD % signal change	0.04	−0.54 – 0.63	0.88	−0.07	−0.42 – 0.28	0.69

B = standardized beta, BOLD = Blood oxygenation level-dependent, CGM = cortical grey matter, CI95 = 95% confidence interval, NAWM = normal-appearing white matter, PSMD = peak width of skeletonized mean diffusivity, Qflow = quantitative flow (velocity phase contrast MRI).

### 3.2 Voxel-wise correlations between endothelial-independent vascular reactivity and MD

**Fig. 1:**
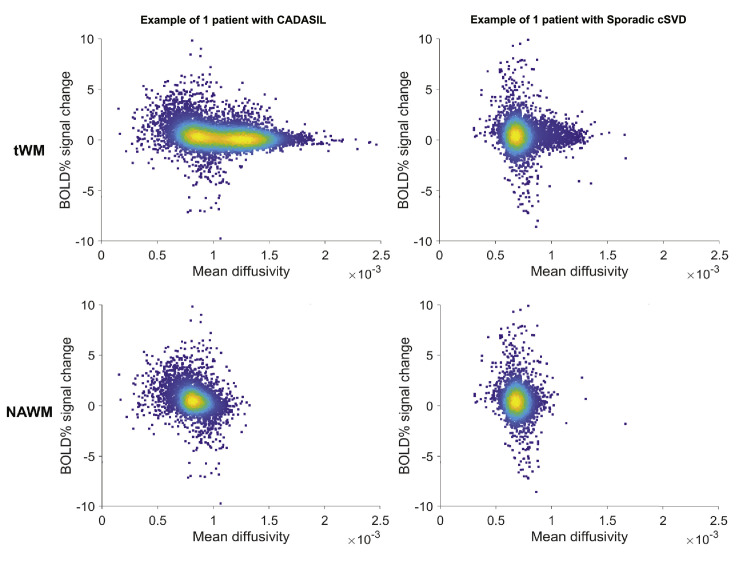
Example density plots for one representative CADASIL and one sporadic cSVD patient.

**Fig. 2:**
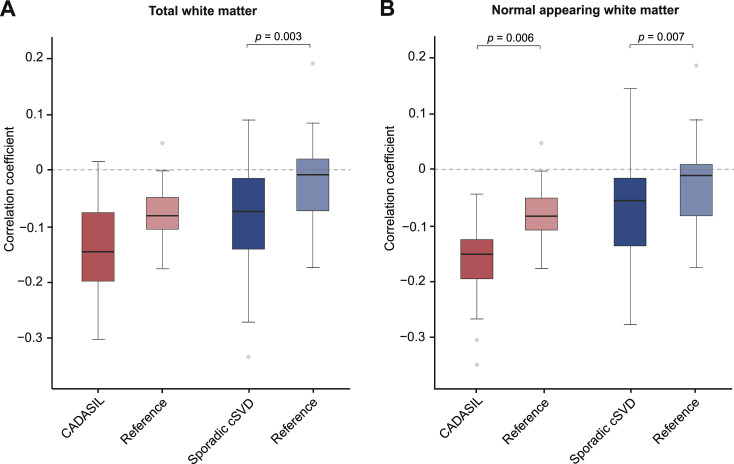
Boxplots showing the pooled correlation coefficients derived from each participant's voxel-wise relation between BOLD % signal change to hypercapnia and mean diffusivity in (A) total white matter and (B) normal appearing white matter.

In voxel-wise analyses, aimed at addressing the second research aim, we calculated the correlation coefficient between BOLD % signal change and MD across all voxels within the white matter mask. In Fig. 1 we show an example density plot of what this correlation looks like for all the voxels of one single patient with CADASIL and for all the voxels of one single patient with sporadic cSVD, both in the total WM and NAWM. These correlations were then pooled for each group. The within group analyses showed a negative correlation between vascular reactivity to hypercapnia and MD across voxels in the white matter. This significant negative correlation was found both in patients with CADASIL (pooled individual correlation coefficients mean(r)±sd: −0.14±0.08; Wilcoxon rank sum test *p* < 0.0001, indicating that a significant proportion of patients showed this negative correlation) and in patients with sporadic cSVD (pooled *r* = −0.10 ± 0.09; Wilcoxon *p* < 0.0001), as visualized in Fig. 2. The pooled correlation coefficient in controls was significantly negative in the CADASIL reference group (pooled *r* = −0.10 ± 0.07; Wilcoxon *p* = 0.005), but not in the sporadic cSVD reference group (pooled *r* = −0.02 ± 0.06; Wilcoxon *p* = 0.21).

Each panel shows the relationship at voxel level between BOLD % signal change and mean diffusivity in total white matter (tWM; upper panels) and normal-appearing white matter (NAWM; lower panels) including all WM voxels from these regions of interest for a single patient with CADASIL (left panels) and a single patient with sporadic cSVD (right panels). For these two exemplar patients, each dot in this density plot thus represents the voxel level relation of the BOLD % signal change and mean diffusivity for all voxels included for that patient. Note that the main difference between the tWM and the NAWM plots is that the tWM plots show a tail of voxels with higher mean diffusivity extending to the right involving WMH, which are omitted from the NAWM plots. From these voxel-wise correlations, we calculated a single correlation coefficient between BOLD % signal change and mean diffusivity for each participant.

The boxplot shows the correlation coefficients that were calculated for each individual participant for the voxel-wise relation between BOLD % signal change and mean diffusivity per participant across all WM voxels (both in patient and reference groups). We tested whether the mean of these pooled individual correlation coefficients was different between the patient groups and their respective reference groups. The negative associations are significantly stronger for the patient groups, except for the difference between CADASIL and the reference group in total white matter (see difference between light-coloured and dark-coloured boxes), reflecting voxels with worse CVR (i.e. lower bold signal change) having worse white matter integrity (i.e. higher MD), see scatterplots for exemplar patients in Fig. 1. * indicates *p* < 0.05.

This pooled negative correlation was stronger in the patient groups than in their respective reference groups, both in the total white matter (mean difference CADASIL and reference group −0.04, *p* = 0.3; mean difference sporadic cSVD and reference group −0.08, *p* = 0.003; Fig. 2) and NAWM (mean difference CADASIL and reference group 0.01, *p* = 0.006, mean difference sporadic cSVD and reference group 0.06, *p* = 0.007; Fig. 2).

## 4 Discussion

This study shows that dysfunction of the small cerebral vessels is significantly associated with white matter tissue alterations both on a whole-brain and, within subjects, also at a local level. This confirms our hypotheses that abnormalities in small vessel function relate to decreased white matter integrity and that local variance in vessel dysfunction relates with local variance in white matter integrity within individual patients. These associations were evident both in patients with CADASIL and sporadic cSVD, although possibly with a different vascular signature, involving perforating arteries in the centrum semiovale in CADASIL and in the basal ganglia in sporadic cSVD.

We found regional and disease-specific differences in the relationship between blood flow velocity and pulsatility in perforating artery populations of the brain and whole-brain white matter microstructure. In patients with CADASIL, decreased white matter integrity was related to decreased blood flow velocity in perforating arteries in the centrum semiovale, while in patients with sporadic cSVD a comparable association was found for blood flow velocity in perforating arteries in the basal ganglia. These vessel- and disease-specific associations are in accordance with our earlier findings of increased WMH volume being related to decreased blood flow velocity in perforating arteries in the centrum semiovale in CADASIL and with decreased blood flow velocity in perforating arteries in the basal ganglia in sporadic cSVD [14,15]. The current results add that decreased blood flow velocity in these perforating artery populations also relates with more subtle microstructural changes in the white matter as measured with PSMD, even in the white matter that appears normal on conventional FLAIR and T1-weighted imaging. In addition, an autopsy study in CADASIL showed similar regional differences, reporting an unchanged lumen diameter in the basal ganglia, but a decreased lumen diameter in perforating arteries in the centrum semiovale due to intimal thickening and fibrosis [34]. Together, these findings indicate that different small vessel populations are primarily affected in CADASIL versus sporadic cSVD and that this likely reflects differences in the underlying physiological mechanisms that contribute to parenchymal injury.

We also measured endothelial-dependent and independent vascular reactivity to a visual stimulus and hypercapnic stimulus in different regions of interest in the brain and studied the relationship with whole-brain white matter microstructure. We found some indications of relationships at this whole-brain level, but they were not robust which might partly relate to the relatively small sample size of our study. Given our hypothesis that local variation in vascular dysfunction explains regional variance in tissue injury, we additionally performed voxel-wise analyses. At a voxel-level, there was a significant negative correlation between endothelial-independent reactivity and white matter microstructure within both patient groups, reflecting a link between decreased reactivity and decreased microstructural white matter integrity. This link was stronger in patients than in their respective reference groups, suggesting this is a disease-related phenomenon rather than part of normal physiology. Furthermore, the correlation between decreased reactivity and decreased white matter integrity was not only apparent in total white matter but also in NAWM, which, in line with earlier research [35], suggests that disease processes linking vascular reactivity and white matter integrity are already taking place in tissue that looks healthy on conventional MRI scans. It can be argued that the individual participant correlation coefficients as summarized in Fig. 2 are small. Yet, it would not be expected a priori that a measure like vascular reactivity is the primary determinant of variance in MD in individual voxels, first and foremost because this variance is not only determined by tissue injury, but also by innate tissue features. The observation that we find a consistent negative association between vascular reactivity and white matter integrity across patients supports a biologically relevant signal. Our results are in accordance with earlier studies on 3T-MRI that have consistently reported an association between whole-brain decreased vascular reactivity and increased whole-brain WMH burden and white matter alterations, both in patients with CADASIL and sporadic cSVD [[6], [7], [8], [9], [10], [11], [12], [13]]. Our findings combined with previous findings on 3T MRI indicate a link between vessel dysfunction and white matter tissue alterations. Given that the voxel-wise analyses from our current study eliminate the influence of confounders, our findings provide a more direct lead towards a possible causal relation between decreased white matter endothelial-independent reactivity and decreased white matter microstructure integrity, both in patients with CADASIL and with sporadic cSVD.

The main strength of this study is that we used state-of-the-art 7T measures of small vessel function, which measure complementary aspects of function from distinct vessel populations. These measures provided the opportunity to assess both whole-brain relationships and voxel-wise ones, which are free of confounders (i.e. shared risk factors for abnormal small vessel function and cerebral tissue injury). Importantly, all participants were scanned on the same 7T MRI scanner in Utrecht. Furthermore, we were able to study both a genetically-defined and thus “purer”, as well as a sporadic form of cSVD. Moreover, the use of two independent cohorts shows the consistency of the results, while also confirming the interesting insight that differential vessel populations with altered vessel function are involved in CADASIL versus sporadic cSVD. A limitation of the study is the relatively small sample size of both patient groups that also imposed restrictions on rigorous control for repeated testing. Furthermore, there is selection bias in our CADASIL patient group as they were required to travel internationally to be included in the study, leaving out the more affected patients. However, we would expect disease effects to be more pronounced in more affected patients, meaning that we would have expected even stronger relations. We also need to be aware that, even though we carefully removed the larger draining veins from our analyses, the BOLD signal on 7T is likely still influenced by signal from smaller draining veins as well. Medication use, particularly of antihypertensives, statins and antiplatelets, is inherently different in patients with cSVD versus control participants, which could have affected some of the relations we report. However, interestingly, no significant relations between the small vessel function measures on 7T-MRI with antihypertensive, statin or antiplatelet use were found in our earlier reports [14,15]. Also, the within participant voxel-wise analyses that we performed are not influenced by medication use. In our voxel-wise analyses, we could only assess reactivity to hypercapnia in the white matter in relation to loss of white matter microstructure, because this is the only small vessel function measure that we acquired on voxel level. For the CADASIL patient group, multi-shell diffusion data was acquired, which would have allowed for more complex modelling such as diffusion kurtosis and biophysical diffusion models. However, this data was not available in the sporadic cSVD group and we decided to keep the analysis harmonized between the two groups by using only the tensor model. Lastly, we only had cross-sectional data available for this study and longitudinal data is needed to establish causality in the relationship between small vessel function and tissue injury; such studies are currently underway [13].

## 5 Conclusion

This study reports global and local associations between multiple measures of small vessel dysfunction and white matter tissue alterations, both in CADASIL and sporadic cSVD. Decreased perforating artery blood flow velocity along different parts of the vascular tree related to decreased white matter integrity and, on a voxel level, decreased small vessel reactivity related to decreased white matter integrity. This shows that abnormalities in small vessel function relate to decreased white matter integrity, even beyond visible lesions. The within subject results on a voxel level, in particular, provide an indication that decreased small vessel function could causally relate to decreased white matter integrity. Follow-up studies that are necessary to further address this question of causality are underway.

## Funding sources

ZOOM@SVDs is part of SVDs@target that has received funding from the European Union's Horizon 2020 research and innovative program under grant agreement No. 666,881. This work is also supported by Vici Grant 918.16.616 from The Netherlands Organisation for Scientific Research (NWO) to GJB. SP and JS are supported by the University Medical Center Utrecht, Brain Center - Rudolf Magnus Young Talent Fellowship 2019. The research of ADL is partly supported by 10.13039/501100010969Alzheimer Nederland (WE.03–2022–11) and the Hilary and Galen Weston Foundation (UB190097). MDi has received funding from the Vascular Dementia Research Foundation and the LMUExcellent Investitionsfond. This work was also funded by the Deutsche Forschungsgemeinschaft (DFG, German Research Foundation) under Germany's Excellence Strategy within the framework of the Munich Cluster for Systems Neurology (EXC 2145 SyNergy – ID 390,857,198) to AK and MDi.

## CRediT authorship contribution statement

**Naomi Vlegels:** Writing – review & editing, Writing – original draft, Visualization, Methodology, Formal analysis, Conceptualization. **Hilde van den Brink:** Writing – review & editing, Writing – original draft, Visualization, Methodology, Formal analysis, Data curation, Conceptualization. **Anna Kopczak:** Writing – review & editing, Resources, Data curation. **Tine Arts:** Writing – review & editing, Methodology, Investigation, Data curation. **Stanley D.T. Pham:** Writing – review & editing, Software, Methodology, Data curation. **Jeroen C.W. Siero:** Writing – review & editing, Supervision, Methodology, Investigation. **Benno Gesierich:** Writing – review & editing, Methodology, Data curation. **Alberto De Luca:** Writing – review & editing, Methodology, Data curation. **Marco Duering:** Writing – review & editing, Methodology, Investigation, Data curation. **Jaco J.M. Zwanenburg:** Writing – review & editing, Methodology, Data curation. **Martin Dichgans:** Writing – review & editing, Funding acquisition, Data curation. **Geert Jan Biessels:** Writing – review & editing, Writing – original draft, Supervision, Funding acquisition, Conceptualization.

## Declaration of competing interest

The authors declare that there is no conflict of interest.

## Appendix Supplementary materials

Image, application 1
