# Supplementary material for: The relation between cerebral small vessel function and white matter microstructure in monogenic and sporadic small vessel disease - the ZOOM@SVDs study
Source: Cereb Circ Cogn Behav. 2025 Mar 24;8:100383. doi: 10.1016/j.cccb.2025.100383 (PMC11994352; doi:10.1016/j.cccb.2025.100383)
Supplement: Supplementary file 1 [file mmc1.docx]

**Supplemental Material**

|  |  | **CADASIL** | **Reference** | **­** |  | **Sporadic cSVD** | **Reference** |  |
| --- | --- | --- | --- | --- | --- | --- | --- | --- |
|  |  | n = 23 | n = 13 | *p* |  | n = 46 | n = 21 | *p* |
| **2D-Qflow centrum semiovale^a^** |  | n=22 | n=10 |  |  | n=46 | n=21 |  |
| Blood flow velocity [cm/s] |  | 0.54±0.06 | 0.63±0.13 | **0.03** |  | 0.65±0.12 | 0.65±0.10 | 0.87 |
| Pulsatility index^b^ |  | 0.56±0.19 | 0.37±0.11 | **0.009** |  | 0.35±0.13 | 0.32±0.11 | 0.34 |
| **2D-Qflow basal ganglia** |  | n=21 | n=9 |  |  | n=44 | n=21 |  |
| Blood flow velocity [cm/s] |  | 3.07±0.67 | 4.05±0.83 | **0.003** |  | 3.7±0.69 | 3.9±0.70 | 0.35 |
| Pulsatility index^b^ |  | 0.46±0.12 | 0.29±0.15 | 0.06 |  | 0.45±0.14 | 0.36±0.13 | **0.005** |
| **BOLD visual stimulus** |  | n=19 | n=10 |  |  | n=35 | n=19 |  |
| BOLD% signal change |  | 0.61±0.20 | 0.82±0.25 | **0.04** |  | 0.63±0.2 | 0.66±0.16 | 0.51 |
| Full-width-at-half-maximum [s] |  | 3.82±0.65 | 3.94±0.36 | 0.60 |  | 3.37±0.97 | 4.01±0.81 | **0.02** |
| **BOLD hypercapnic stimulus^cd^** |  | n=17 | n=11 |  |  | n=36 | n=18 |  |
| CGM BOLD% signal change |  | 3.66±1.24 | 3.07±1.20 | 0.26 |  | 3.66±1.38 | 3.39±1.43 | 0.51 |
| tWM BOLD% signal change |  | 0.35±0.33 | 0.17±0.31 | 0.31 |  | 0.56±0.44 | 0.55±0.38 | 0.96 |

**Supplementary Table 1. Small vessel function in patients with CADASIL and sporadic cSVD and their respective reference groups.**

^a^ The region of interest is the entire semioval centre and basal ganglia excluding lacunes. Analyses are corrected for age and sex. ^b^ Additional correction for blood flow velocity. ^c^ Analyses corrected for age, sex and change in end-tidal CO_2_ to hypercapnia. ^d^ Vascular reactivity to hypercapnia was reduced in WMH (compared to NAWM in both CADASIL: mean BOLD% change difference -0.29, *p* = 0.02 and sporadic cSVD: mean BOLD% change difference -0.35, *p* < 0.001).

BOLD = Blood oxygenation level-dependent, CGM = cortical grey matter, Qflow = quantitative flow (velocity phase contrast MRI), tWM = total white matter

|  | **Diffusion** |  |  |
| --- | --- | --- | --- |
| **7T small vessel function measures** | **Total WM  PSMD** | **Total WM  MD per voxel** | **NAWM  MD per voxel** |
| **Centrum semiovale – 2D-Qflow** |  |  |  |
| Blood flow velocity | x |  |  |
| Pulsatility index | x |  |  |
| **Basal Ganglia – 2D-Qflow** |  |  |  |
| Blood flow velocity | x |  |  |
| Pulsatility index | x |  |  |
| **Visual Cortex – BOLD visual stimulus** |  |  |  |
| BOLD% signal change | x |  |  |
| Full width at half maximum [s] | x |  |  |
| **Whole brain – BOLD hypercapnic stimulus** |  |  |  |
| Cortical GM BOLD% signal change | x |  |  |
| Total WM BOLD% signal change |  | x | x |
| NAWM BOLD% signal change | x |  |  |

**Supplementary Table 2. Overview of measures per brain region and investigated associations indicated with an x.**

BOLD = Blood oxygenation level-dependent, GM = grey matter, NAWM = normal-appearing white matter, PSMD = peak width of skeletonized mean diffusivity, Qflow = quantitative flow (velocity phase contrast MRI), WMH = white matter hyperintensities.

|  |  | **PSMD WM (log transformed)** | | |  | | **PSMD NAWM (log transformed)** | | | |
| --- | --- | --- | --- | --- | --- | --- | --- | --- | --- | --- |
|  |  | B | CI95 | *P* | |  | | B | CI95 | *p* |
| **2D-Qflow centrum semiovale** |  | N= 46 |  |  | |  | | N = 46 |  |  |
| Blood flow velocity [cm/s] |  | 0.07 | -0.20 – 0.34 | 0.61 | |  | | 0.08 | -0.20 – 0.36 | 0.58 |
| WM Pulsatility Index |  | 0.09 | -0.18 – 0.36 | 0.51 | |  | | 0.08 | -0.21 – 0.37 | 0.58 |
| **2D-Qflow basal ganglia** |  | N = 44 |  |  | |  | | N = 44 |  |  |
| Blood flow velocity [cm/s] |  | -0.32 | -0.60 – -0.04 | **0.03** | |  | | -0.23 | -0.51 – 0.06 | 0.12 |
| Pulsatility index |  | 0.19 | -0.09 – 0.48 | 0.18 | |  | | 0.18 | -0.10 – 0.46 | 0.21 |
| **BOLD visual stimulus** |  | N = 35 |  |  | |  | | N = 35 |  |  |
| BOLD% signal change |  | -0.05 | -0.36 – 0.25 | 0.72 | |  | | 0.01 | -0.28 – 0.29 | 0.96 |
| Full-width-at-half-maximum [s] |  | -0.25 | -0.54 – 0.05 | 0.10 | |  | | -0.17 | -0.45 – 0.10 | 0.21 |
| **BOLD hypercapnic stimulus** |  | N = 36 |  |  | |  | | N = 36 |  |  |
| CGM BOLD% signal change |  | -0.28 | -0.59 – 0.03 | 0.07 | |  | | -0.28 | -0.58 – 0.02 | 0.07 |
| SGM BOLD% signal change |  | -0.24 | -0.55 – 0.07 | 0.12 | |  | | -0.21 | -0.51 – 0.10 | 0.18 |
| WMH BOLD% signal change |  | -0.30 | -0.62 – 0.03 | 0.07 | |  | | -0.29 | -0.62 – 0.04 | 0.08 |
| NAWM BOLD% signal change |  | -0.03 | -0.34 – 0.29 | 0.87 | |  | | -0.09 | -0.40 – 0.22 | 0.58 |

**Supplementary Table 3. Linear regressions between 7T small vessel function measures and PSMD corrected for age in patients with sporadic cSVD**

B = standardized beta, BOLD = Blood oxygenation level-dependent, CGM = cortical grey matter, CI95 = 95% confidence interval, NAWM = normal-appearing white matter, PSMD = peak width of skeletonized mean diffusivity, Qflow = quantitative flow (velocity phase contrast MRI), SGM = subcortical grey matter.
